# Supplementary figures and images for: Satellite tagging highlights the importance of productive Mozambican coastal waters to the ecology and conservation of whale sharks
Source: PeerJ. 2018 Jan 2;6:e4161. doi: 10.7717/peerj.4161 (PMC5755488; doi:10.7717/peerj.4161)

**A**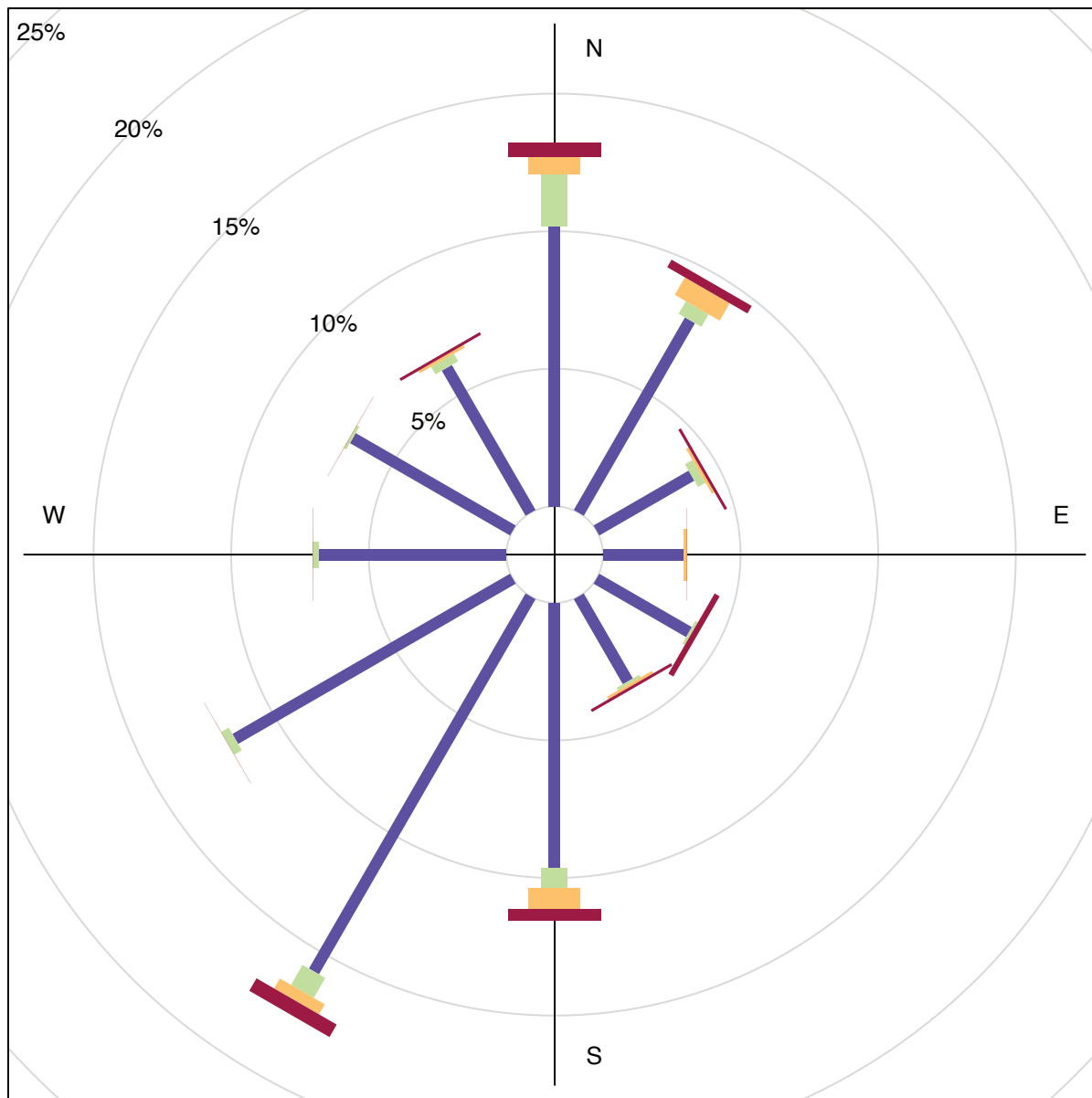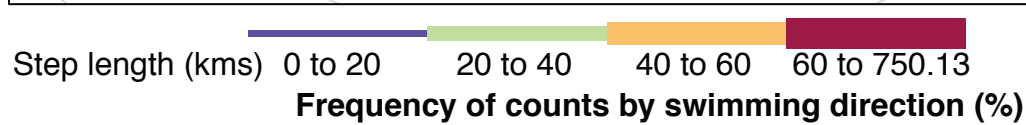**B**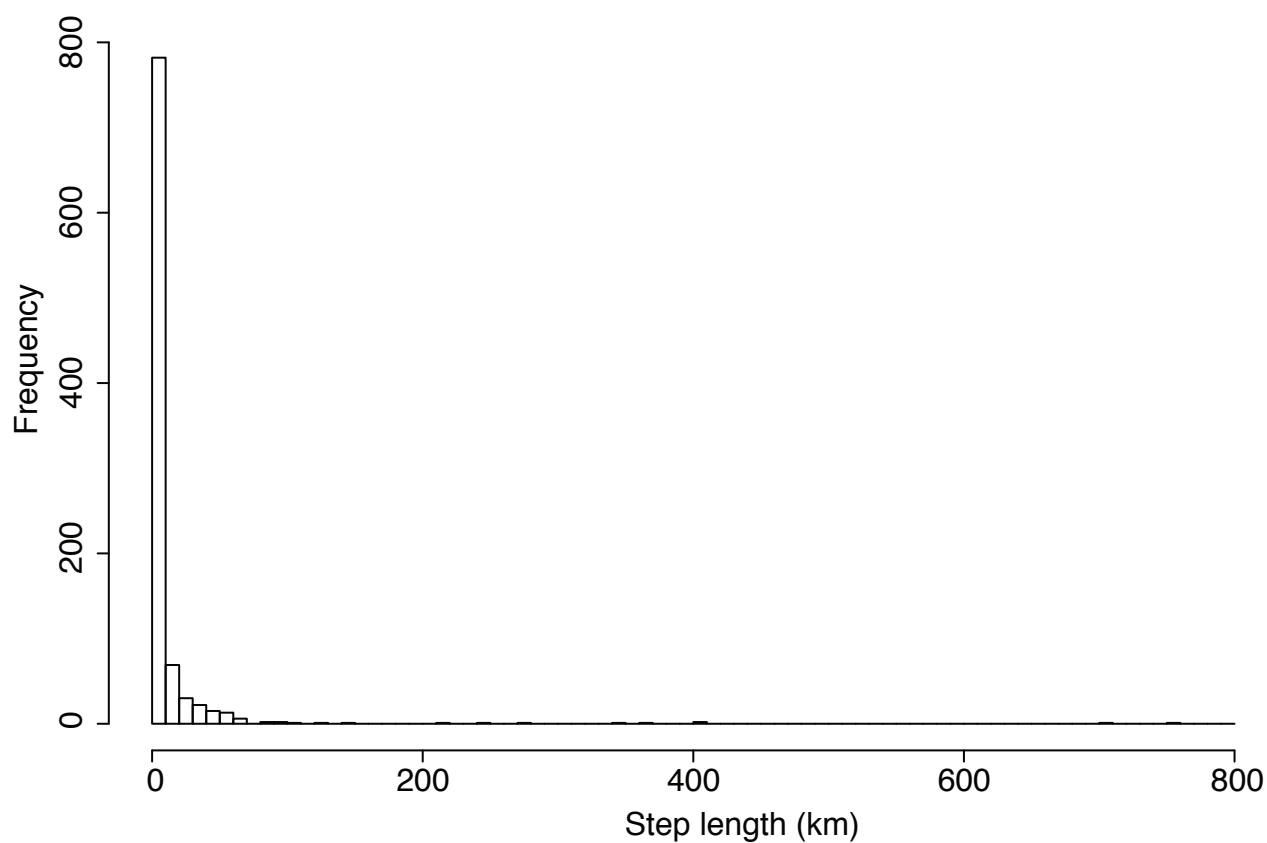

Supplement: Figure S1 — (A) Frequency of directions and (B) the step length frequency for tagged whale sharks. [file peerj-06-4161-s001.pdf]
